# *Klrb1* Loss Promotes Chronic Hepatic Inflammation and Metabolic Dysregulation

**DOI:** 10.3390/genes15111444

**Published:** 2024-11-08

**Authors:** Shuqi Yang, Tingting Luo, Haoran Liu, Li Chen, Jinyong Wang, Yongju Zhao, Xuemin Li, Haohuan Li, Mingzhou Li, Lu Lu

**Affiliations:** 1 State Key Laboratory of Swine and Poultry Breeding Industry, College of Animal Science and Technology, Sichuan Agricultural University, Chengdu 610000, China; 2022302137@stu.sicau.edu.cn (S.Y.); luotingting@stu.sicau.edu.cn (T.L.); 202100472@stu.sicau.edu.cn (H.L.); lixuemin@stu.sicau.edu.cn (X.L.); lihaohuan@sicau.edu.cn (H.L.); 2 Department of Pig Production, Chongqing Academy of Animal Science, Chongqing 400000, China; lichen5696@163.com (L.C.); kingyou@vip.sina.com (J.W.); 3 College of Animal Science and Technology, Southwest University, Chongqing 400000, China; zyongju@163.com

**Keywords:** Klrb1, immune, inflammation, RNA-seq, DEGs, metabolism

## Abstract

**Background/Objectives:** CD161, encoded by the *KLRB1* gene, is an inhibitory receptor expresses on various immune cell and has gained attention in immune checkpoint research. In recent studies, *KLRB1* has been found to be one of the potential markers of liver diseases such as cirrhosis. Therefore, it will be important to understand what process *KLRB1* involved in the liver for the prevention of liver diseases. **Methods:** We compared *KO* mice with wild-type controls by routine blood analysis and RNA-seq, and additionally performed H&E staining and qPCR to validate the differentially expressed genes (DEGs). **Results:** *KO* mice had fewer lymphocytes compared to the wild-type mice. A transcriptomic analysis showed that *Klrb1* loss causes the upregulation of immune-related genes and pathways like NOD-like receptor and p53 signaling, while causing the downregulation of lipid metabolism-related genes. A protein interaction analysis indicated a potential cancer risk under chronic inflammation. Histological examination with H&E staining reveals an inflammatory response around the central venous vessels in the liver tissue of the *KO* mice. **Conclusions**: We conclude that *Klrb1* knockout disrupts the immune and metabolic functions in the liver, which may possibly lead to chronic inflammation and malignancy risks. These findings highlight the role of *Klrb1* in hepatic health.

## 1. Introduction

CD161, also known as NK receptor-P1A (NKRP1A), is a member of the killer cell lectin-like receptor B subfamily member 1 (KLRB1) [1]. In humans, CD161 is encoded by a single gene *KLRB1* [2,3], which is expressed in many types of immune cells, while CD161 expression can be detected in most NK cells [4]. *CLEC2D* mRNA is expressed in both malignant cells and immunosuppressive myeloid cells [5]. LLT1/CD161 interaction plays an important role in the regulation of immune responses and cell activation in infectious diseases, autoimmunity, inflammation, and cancer.

NK cells play an important role in the pathogenesis of liver disease, acting as immune sentinels that recognize and eliminate virus-infected and tumor cells while also activating other immune cells through the secretion of cytokines to enhance immune responses. However, in chronic liver disease and liver cancer, NK cells often exhibit functional dysregulation, leading to reduced cytotoxicity and cytokine secretion, which in turn promote liver damage and exacerbate inflammation [6]. Therefore, a deeper understanding of the functional changes in NK cells not only aids in comprehending the pathological processes of liver disease but also provides potential targets for developing new immunotherapeutic strategies [7].

The CD161 receptor encoded by the Klrb1 gene is expressed in various NK/ILC and T cell subsets circulating throughout the body in mammals. Kirkham CL et al. used RT-PCR to verify the expression of Klrb1 in different tissues of mice, and the results indicated that the transcript Nkrp1 is expressed in the spleen, thymus, lymph nodes, and other hematopoietic tissues, correlating with the existence of NK cells in these organs [8]. Meanwhile, the CD161 receptor is also expressed in various immune cells [4], and low levels of LLT1 are found in the hepatocytes of healthy livers [9]. The CD161/LLT1 receptor–ligand interaction participates in the regulation of immune responses in the liver through the bloodstream.

Existing studies have shown that KLRB1 is associated with various types of tumors, and CD161 has become one of the hot spots of research into immunotherapy targets in recent years, in the malignant tissues of testicular germ cell tumors (TGCTs) [10], pancreatic ductal adenocarcinoma [11], HPV-positive oropharyngeal cancer [12], and diabetic non-small cell lung cancer [13]. Most recently, a few studies have reported the correlation of KLRB1 with hepatic diseases. Nasr Azadani et al. performed RNA-seq transcriptome analysis of liver samples from patients with Hepatitis C virus (HCV)-related cirrhosis and identified *LTB*, *ZAP70*, *KLRB1*, *ISLR*, *MOXD1*, and *Slitrk3* as promising biomarkers for diagnosing HCV-related cirrhosis [14]. Siting Fang et al. used TCGA data and performed a single-cell analysis on them, evaluating the diagnostic performance of *KLRB1* on NK and CD8^+^T cells in peripheral blood samples from 126 hepatocellular carcinoma (HCC) patients. They found that *KLRB1* expression in NK and T cells in HCC patients was downregulated compared with healthy individuals, and believed that *KLRB1* could become a prognostic marker for HCC diagnosis [15]. Research by Nathan D. Mathewson and colleagues indicates that the inactivation of the CD161 receptor mediated by the *KLRB1* gene enhances the cytotoxicity of T cells against glioma cells in vitro, as well as their anti-tumor function in vivo [16].

Therefore, figuring out what role *KLRB1* plays in the liver can help us further understand liver-related diseases. There are already articles that have analyzed cancerous liver tissues using RNA-seq [17], as well as studies that have identified differentially expressed genes through RNA-seq and proposed them as potential candidate targets for treating non-alcoholic fatty liver disease (NAFLD) [18].

Given the abovementioned reasons, we aimed to reveal the basic role of *Klrb1* in the liver under normal conditions by comparing *Klrb1* knockout mice with wild-type mice. This study found that the inactivation of *KLRB1* makes the hematopoietic function of mice abnormal, and the livers of these mice showed chronic inflammation and metabolic disorders. This study believes that *KLRB1* might be used as a predictive checkpoint for chronic liver disease to provide a theoretical basis and indirect reference for the role of *KLRB1* in the development of immune checkpoint inhibitors.

## 2. Materials and Methods

### 2.1. Animals

C57BL/6JGpt-Klrb1^em1Cd^/Gpt (*KO*) mice and C57BL/6JGpt mice (WT) were purchased from GemPharmatech company (https://www.gempharmatech.com/, accessed on 28 October 2024) and housed in an animal facility compliant with experimental animal management regulations. The facility was maintained at a temperature of 20–24 °C, with a relative humidity of 40–60% and a light/dark cycle of 12 h of light and 12 h of dark. The mice were fed a standard, commercially available rodent diet and provided with filtered water, both of which were changed daily. After the mice had acclimated to the housing conditions for 1 month, 5 *KO* mice and 9 WT were euthanized by cervical dislocation. Following this, 0.5 mL of blood was collected from the retro-orbital venous plexus and stored in EDTA anticoagulant tubes for routine blood tests, and a portion of the liver was collected and preserved in 4% paraformaldehyde, and the remaining tissue was used for further RNA sequencing and RT-PCR. The experiments were conducted according to these protocols and were approved by the Animal Ethics Committee of Sichuan Agricultural University under license number 20220393 (approval date: 14 September 2022).

### 2.2. Hematoxylin and Eosin Staining

We prepared histological sections and performed HE staining on samples from three WT groups and four *KO* groups for comparison. During the sample preparation process, fresh tissue was first fixed in a fixing solution for more than 24 h. The target tissue was then trimmed with a surgical knife and placed in a dehydration box for dehydration, and sequentially treated with varying concentrations of alcohol, anhydrous ethanol, xylene, and wax. Next, the wax-embedded tissue was embedded in an embedding machine, cooled, and then removed and trimmed into wax blocks. The wax blocks are cut into 3 μm thick sections, which were then floated in warm water to flatten them and dried in an oven at 60 °C. After dewaxing and staining with hematoxylin and eosin, the sections underwent further dehydration and a transparency treatment before being mounted with neutral gum. Finally, images of the sections were captured for analysis using an optical microscope (NIKON Eclipse SI, Melville, NY, USA).

### 2.3. Routine Blood Test

The following parameters were then analyzed using a VetScan HM5 (Abaxis, Union City, CA, USA) automated hematology analyzer: monocyte percentage (Mon%), red blood cells (RBCs), hematocrit (HCT), mean corpuscular volume (MCV), mean corpuscular hemoglobin (MCH), mean corpuscular hemoglobin concentration (MCHC), mean platelet volume (MPV), hemoglobin (HGB), lymphocyte percentage (Lymph%), lymphocytes (Lymph), white blood cells (WBCs), monocytes (Mon), red blood cell distribution width (RDW), platelet distribution width (PDW), platelet count (PCT), platelets (PLTs), granulocyte percentage (Gran%), and granulocytes (Gran).

### 2.4. Total RNA Isolation, Bulk RNA-Seq, and RT-PCR

We extracted tissue samples using the Trizol method, and all RNA was assessed using an Agilent 2100 bioanalyzer (Santa Clara, CA, USA). RNA samples with optical densities between 1.8 and 2.0 were selected for Bulk RNA-seq sequencing. The sequencing of 14 samples was conducted on the Novagene Company’s Illumina NovaSeq 6000 platform (Beijing, China), yielding a total of 104.51 G of raw data and 92.32 G of clean data after filtering. The RNA-seq data were submitted to the Genome Sequence Archive (GSA) database: CRA019416. The clean data were aligned using STAR software on a Linux system, version 7.4.1708 (Core). We first downloaded the mouse reference genome (Mus_musculus.GRCm39.DNA.toplevel.fa.gz) from Ensembl (https://ftp.ensembl.org/pub/release-111/fasta/mus_musculus/, accessed on 5 March 2024) to construct the genome index. RNA-seq reads were then aligned to the genome using STAR, resulting in alignment rates and related information.

Next, we quantified the clean data using Kallisto (0.50.1) software, constructing an index with the Mus_musculus transcripts (Mus_musculus.GRCm39.111.gtf.gz) obtained from the Ensembl website (https://ftp.ensembl.org/pub/release-111/gtf/mus_musculus/, accessed on 5 March 2024) for pseudo-alignment and integration. Following the quantification of the whole transcriptomes of *KO* mice and their wild-type counterparts, we normalized the liver gene expression data, resulting in TPM gene expression matrices and count matrices for both groups. Subsequently, we performed a t-SNE analysis on the transcriptomic data to examine the relationships among samples. We used edgeR to identify differentially expressed genes (DEGs) and conducted a Gene Ontology Enrichment Analysis (GO) and Kyoto Encyclopedia of Genes and Genomes (KEGG) pathway enrichment analysis on these DEGs. Additionally, we performed Gene Set Enrichment Analysis (GSEA) on the overall gene counts for validation.

In the cDNA transcription and RT-PCR experiments, we used Novozymes’ HiScript^®^ III RT SuperMix for qPCR (+gDNA wiper) and Taq Pro Universal SYBR qPCR Master Mix. After RNA-seq analysis, we selected seven DEGs for RT-PCR validation, specifically the upregulated genes *Eci2*, *Cnbd2*, and *Chd9* and the downregulated genes *Nectin1*, *Entpd4*, *Insig1*, and *Midip1*. The primer sequences for these genes can be found in Table S1.

### 2.5. Data Statistics

This study used Prism 10.0 (GraphPad Software, La Jolla, CA, USA) for non-parametric tests. Technical replicates and biological replicates for the RT-PCR were each set at 3; biological replicates for blood routine tests and RNA-seq included the WT group (*n* = 9) and *KO* group (*n* = 5). For HE staining, the biological replicates were the WT group (*n* = 3) and *KO* group (*n* = 4). The results were expressed as means ± standard deviation, with *p* < 0.05 (*) and *p* < 0.01 (**) as the significance levels.

## 3. Results

### 3.1. Inflammatory Response upon Klrb1 Knockout

**Figure 1:**
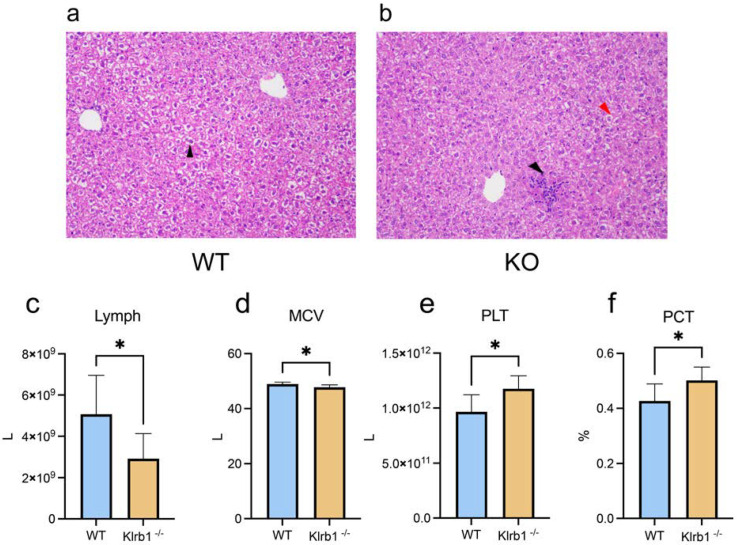
HE staining and routine blood test of *KO* mice and wild-type mice. (**a**) HE staining of WT group; (**b**) HE staining of KO group; (**c**), lymphocyte count (Lymph); (**d**) mean corpuscular volume (MCV); (**e**) platelets (PLTs); (**f**) platelet count (PCT). *p* < 0.05 (*).

To investigate whether *Klrb1* knockout induces inflammatory responses in mice, we prepared histological sections and performed HE staining on samples from three WT groups and four *KO* groups for comparison. The WT group exhibited hepatocyte degeneration characterized by severe cytoplasmic vacuolation and a broader extent, as indicated by a black arrow (Figure 1a). In contrast, the *KO* group showed signs of an inflammatory response around the central vein, with notable neutrophil infiltration (also indicated by a black arrow). Cellular degeneration and vacuolation were milder in the *KO* group, as highlighted by a red arrow (Figure 1b).

Additionally, to explore the physiological effects on KO group, we conducted routine blood analyses to assess changes in the lymphocyte compartment. The results indicated that *KO* mice had a significant decrease in lymphocyte counts and average red blood cell volume compared to wild-type mice (Figure 1c,d). Conversely, there were significant increases in the platelet count and platelet volume in the *KO* group (Figure 1e,f). No significant changes were observed in the remaining indicators (Supplementary Figure S1).

### 3.2. Transcriptome Analysis and Gene Expression

**Figure 2:**
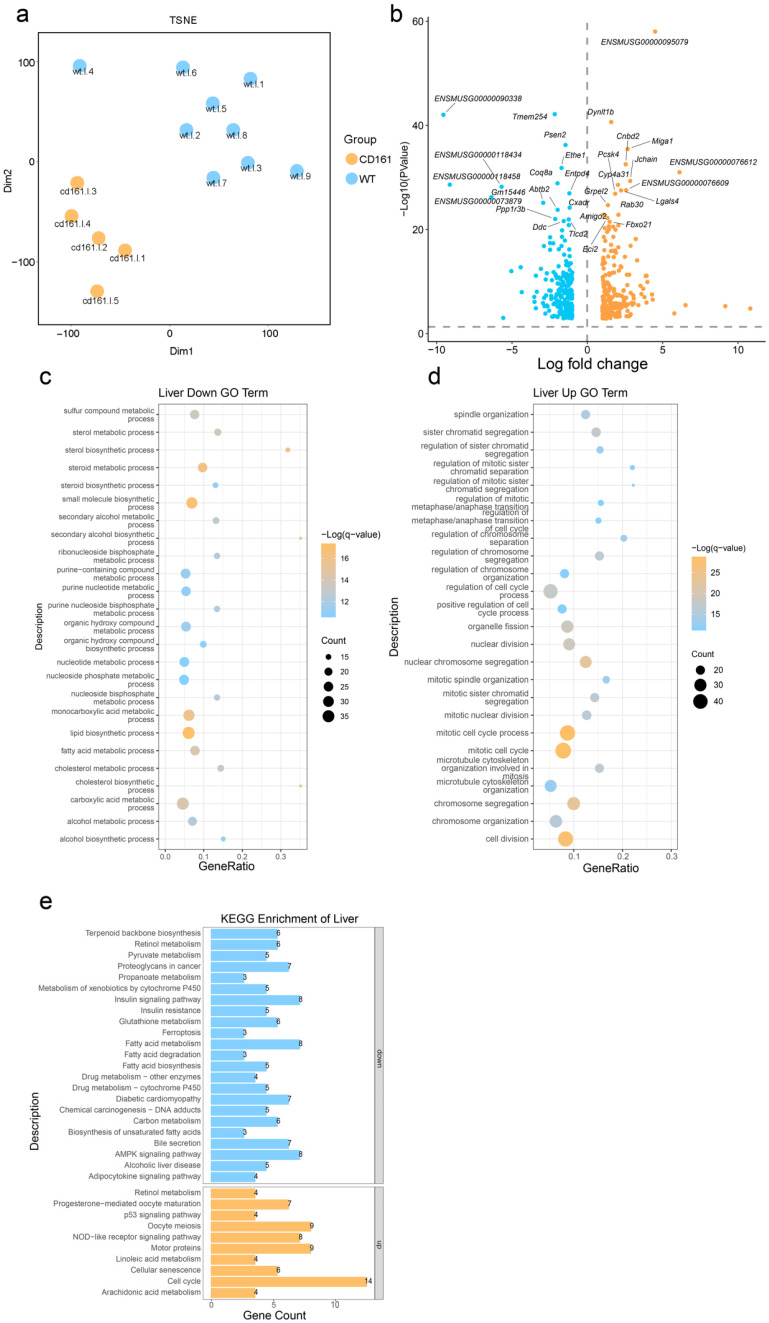
DEG analyses of *KO* mice and wild-type mice. (**a**) t-SNE plot representing the clustering of samples based on gene expression data from the KO group and WT group; (**b**) volcano plot of DEGs; (**c**) top 25 GO terms of the downregulated DEGs; (**d**) top 25 GO terms of the upregulated DEGs; (**e**) bar plot of DEGs.

After data filtering, we obtained hepatic RNA-seq samples from five *KO* mice and nine WT mice containing an average of 10 G in their sequencing depth. T-SNE (t-distributed Stochastic Neighbor Embedding) is a dimensionality reduction technique used for visualizing high-dimensional data by assessing the similarity between data points with a Gaussian distribution and mapping them onto a lower-dimensional space using a t-distribution. This approach helps preserve the relationships between data points and enhances visual interpretability [19]. Therefore, we performed a dimensionality reduction analysis using t-SNE via the Rtsne package to effectively visualize our high-dimensional data. As shown in (Figure 2a), 14 RNA-seq samples were well clustered into two groups, the *KO* and WT groups, indicating that *Klrb1* knockout causes an overall hepatic transcriptome change.

From the counts matrix, we detected a total of 36,106 expressed genes, from which 21,517 protein-coding genes were identified. A differential gene expression analysis was performed using the edgeR package in R, with the thresholds of |Log2FC| ≥ 1 and FDR < 0.01. After normalizing the input gene expression values, we visualized the significantly expressed genes using the ggplot2 package in R, resulting in the volcano plot in Figure 2b. Statistical analysis revealed 252 significantly upregulated genes and 240 significantly downregulated genes in the liver of *KO* mice compared to their wild-type littermates.

### 3.3. Gene Expression Changes Caused by the Deletion of Klrb1

In order to explore the gene expression changes in mouse liver caused by the deletion of *Klrb1*, we first used edgeR to find the DEGs in the two groups and analyzed them in metascape (https://metascape.org/gp/index.html#/main/step1, accessed on 28 October 2024). KEGG and GO function enrichments were carried out, and the potential biological functions of the DEGs found in the liver were analyzed. After that, we identified 326 GO pathways and 28 KEGG pathways (Figure 2c,d). Firstly, the upregulated DEGs mainly enriched in KEGG/GO pathways related to lipid anabolism, inflammatory response, cell proliferation, apoptosis, and aging, while the downregulated DEGs were mainly enriched in KEGG/GO pathways related to energy synthesis and metabolism.

**Figure 3:**
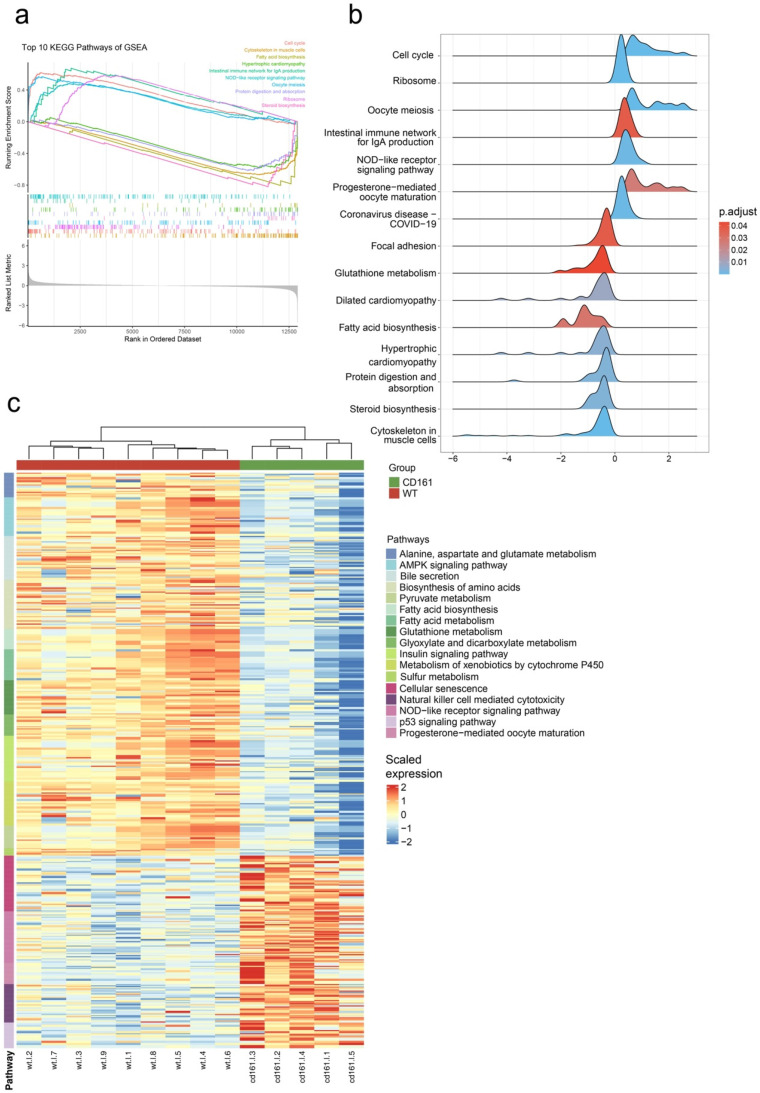
KEGG enrichment of GSEA of *KO* mice and wild-type mice. (**a**) GSEA plot of the top 10 pathways; (**b**) ridge plot of the top 10 pathways; (**c**) heatmap of 17 pathways that we chose.

In addition, to explore the differences in gene expression patterns in the livers of KO mice and wild-type mice, we used the KEGG gene sets from the GSEA to enrich the count matrix of the two groups and selected pathways with the thresholds |NES| > 1, NOM *p*-value < 0.05, and FDR (*padj*) < 0.25 as significantly enriched pathways. Finally, 17 upregulated pathways and 38 downregulated pathways were identified. The top 10 pathways showed that when the *Klrb1* gene was inactivated, the gene expression patterns in the liver exhibited an upregulated trend in processes related to cell proliferation, differentiation, immune response, etc. On the other hand, a downregulated trend was observed in biological processes such as energy metabolism and cell signaling (Figure 3a,b). From the enrichment results, we also found other functions related to metabolism and immunity and selected the gene sets of 17 pathways related to these for display (Figure 3c).

### 3.4. Protein–Protein Interaction of DEGs

**Figure 4:**
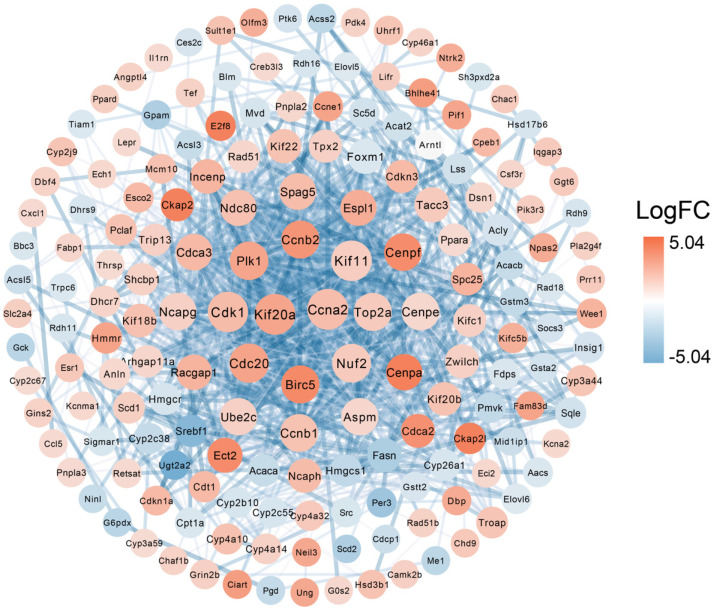
PPI analysis of DEGs. The interaction between each protein pair is represented by a line, and the size of the circle is proportional to the degree of their interaction. Proteins that are closer to the concentric circles have a higher number of interactions with other proteins.

In order to explore the proteins that play a key role in the DEGs between the KO and WT groups, we annotated the DEGs in the STRING database (https://cn.string-db.org/, accessed on 24 March 2024) and finally constructed a PPI network to depict the complex relationships between proteins. The interaction confidence threshold was set to high confidence (0.700), and the k-means clustering method was used to identify and retain the clusters containing the most interrelated genes. The resulting network was visualized using Cytoscape 3.10.3 software, as shown (Figure 4). This method helps to identify hub genes and their associated proteins, thereby gaining insight into the molecular mechanism of liver function changes after *Klrb1* knockout. Calculated by Cytoscape software, the top ten proteins with the highest interaction scores are *Ccna2*, *Kif20a*, *Kif11*, *Cdk1*, *Cdc20*, *Nuf2*, *Plk1*, *Top2a*, *Ccnb2*, *Birc5*, and *Cenpe*.

### 3.5. Validation of DEGs by qPCR

**Figure 5:**
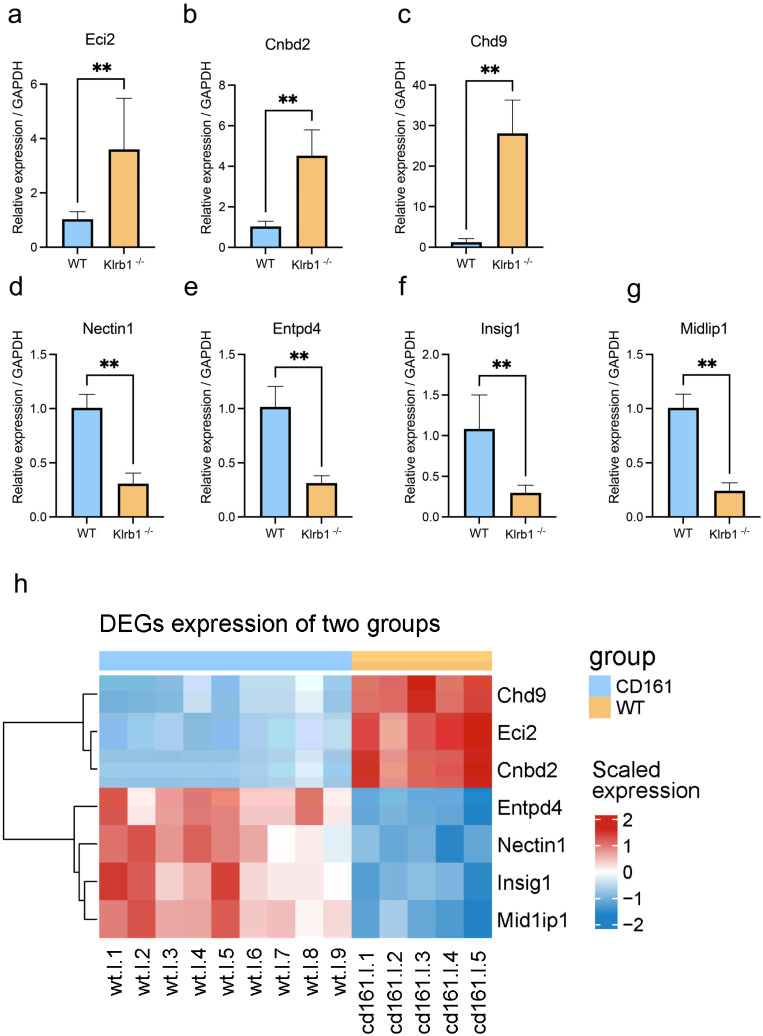
Comparison of differential gene expression between RT-PCR and RNA-seq. (**a**–**g**) Gene expression of DEGs in RT-PCR (*Eci2*, *Cnbd2, Chd9*, *Nectin1*, *Entpd4*, *Insig1*, *Midip1*); (**h**) heatmap of DEGs in RNA-seq (*Eci2*, *Cnbd2, Chd9*, *Nectin1*, *Entpd4*, *Insig1*, *Midip1*); *p* < 0.01 (**).

We performed RT-PCR validation on seven of the DEGs, including the upregulated genes *Eci2*, *Cnbd2*, and *Chd9* and the downregulated genes *Nectin1*, *Entpd4*, *Insig1*, and *Midip1*. Subsequently, we compared the expression levels of these seven genes using RT-PCR and RNA-seq. As shown in Figure 5, the up- and downregulated genes obtained from edgeR also displayed the same trend in the qPCR results, showing their extremely significant expression.

## 4. Discussion

In the context of chronic infection, the hematopoietic system responds to increased immune demands by activating emergency hematopoiesis [20]. This is characterized by the rapid production of neutrophils, monocytes, and dendritic cells in the bone marrow to replenish depleted immune cells. However, an infection-induced disruption of the bone marrow microenvironment may impair its support for lymphopoiesis, leading to a reduction in lymphocyte production [21]. Concurrently, excessive antigenic stimulation can cause T-cell exhaustion [21]. Our findings indicate that lymphocyte counts in *Klrb1* knockout mice were reduced, with their proportion among the white blood cells was significantly lower than that of wild-type mice. In contrast, monocyte proportions were significantly elevated. These results suggest that the absence or dysfunction of the CD161 receptor may lead to the excessive activation of T cells and NK cells, further exacerbating cell exhaustion and increasing susceptibility to bacterial infection.

Additionally, our results show that *KO* mice exhibit a significant reduction in mean red blood cell volume and an increase in red blood cell distribution width, leading us to hypothesize that chronic inflammation may alter hematopoietic dynamics, resulting in the generation of smaller red blood cells. Research has shown that a significant increase in platelets may lead to platelet activation, increasing the risk of blood clotting [22]. Consistently, our results show that *KO* mice had significantly higher platelet counts and platelet volumes than wild-type mice, further suggesting the possibility of coagulation in these mice.

To further explore the molecular mechanisms underlying these phenomena, we performed a transcriptomic analysis to examine the DEGs between *KO* and wild-type mice. The EdgeR analysis revealed a significant number of DEGs, among which several immunoglobulin genes (e.g., *IgA*, *Miga1*, *Ighg2c*, *Jchain*) were significantly upregulated and were within the top 30 DEGs. Previous studies have shown that *IgA* exerts its immune functions by inducing the production of key cytokines by immune cells, which can both induce inflammation and regulate immunosuppressive responses [23]. These genes may be involved in the pathogenesis of various chronic inflammatory diseases by cooperating with other genes. Furthermore, research has indicated that the *NLRP12* gene plays a crucial role in hematopoietic progenitor cells, regulating inflammatory responses by limiting TNF-induced apoptosis [24,25]. In our KEGG enrichment results, upregulated genes were enriched in the NOD-like receptor signaling, cellular senescence, cell cycle, and p53 signaling pathways, suggesting that *Klrb1* inactivation may lead to excessive immune responses in mice, exacerbating antigen-overstimulation-induced apoptosis. This hypothesis was further supported by our GSEA results. In our HE-stained tissue sections, the results indicate an inflammatory response characterized by neutrophil infiltration around the central veins in *KO* group mice. While the neutrophil-related parameters from the routine blood tests did not show significant differences, the observed tissue alterations provide additional evidence supporting our hypothesis. Specifically, the inactivation of the Klrb1 gene may be associated with the presence of an inflammatory response within the mice. This finding underscores the potential role of the Klrb1 gene in modulating inflammatory processes.

Moreover, our study found that *Klrb1* inactivation not only caused immune dysregulation but also induced metabolic disturbances in the liver of mice. KEGG and GSEA enrichment analyses revealed that downregulated DEGs were enriched in pathways related to energy balance regulation, lipid metabolism, and toxin metabolism. Previous studies have demonstrated that adipokines play a critical role in immune regulation [26]. For example, natural killer T (NKT) cells contribute to adipose tissue inflammation and the development of glucose intolerance during diet-induced obesity [27]. Diet-induced obesity also promotes the activation of macrophages in adipose tissue, which enter a pro-inflammatory state and lead to insulin resistance [27]. During inflammation, monocytes can differentiate into inflammatory dendritic cells or macrophages [28]. Therefore, following CD161 receptor inactivation, the reduced lipid metabolism in the liver of *KO* mice may lead to the accumulation of intrahepatic fat, which in turn triggers a pro-inflammatory response, causing liver tissue damage and further impairing hepatic detoxification functions.

Notably, we identified *Amigo2* among the upregulated DEGs. Existing studies have shown that *AMIGO2* serves as a novel marker for liver metastasis in colorectal cancer patients [29]. Furthermore, this finding was supported by the results of the PPI network analysis. While the differences in expression between protein and mRNA levels may not have a strong linear relationship, this approach allows us to narrow down our research focus, helping us identify more critical predictive proteins related to diseases, which is of significant importance [30]. We identified several proteins with high interaction scores (*Cna2*, *Kif20a*, *Kif11*, *Cdk1*, and *Cdc20*) that are closely associated with various cancers [31,32,33,34,35]. Notably, studies have shown that *Cdc20* is highly expressed in the diseased tissues of most hepatocellular carcinoma samples [27]. Therefore, we hypothesize that the liver of *KO* mice may be prone to malignant transformation in the context of chronic inflammation.

This study conducted a preliminary analysis of the liver in *Klrb1*^−/−^ mice at the blood and gene levels using routine blood tests and transcriptome sequencing. Additionally, our qPCR and HE staining results support the reliability of our RNA-seq findings and our hypotheses. The findings suggest that the inactivation of the CD161 receptor has some impact on the immune and metabolic functions of the mouse liver. However, due to the liver’s compensatory and self-healing capabilities, it remains unclear whether these abnormalities will lead to substantial damage in the future. This study confirmed that *Klrb1* is expressed in various immune cells, and since the liver is a key immune regulatory organ, our results indicate that CD161 receptor inactivation affects immune regulation in the liver, along with a disruption in the regulation of energy metabolism. This highlights the close relationship between immune regulation and energy metabolism. However, the specific mechanisms driving these effects are still unclear, and further long-term studies are needed to explore the dynamic expression changes in *Klrb1* and other genes. The current broad investigation into the effects of *Klrb1* on the liver also suggests that lipid metabolism and material metabolism should be considered as future research areas. This paper focuses solely on the effects of *Klrb1* gene inactivation on the liver of female mice. It is worth further exploring whether *Klrb1* inactivation affects male mice and other organ tissues, which may provide clues about how the pathogenesis, prevention, immune checkpoints, and potential drug targets of diseases related to different tissues and organs differ between genders.

## Data Availability

The original data presented in this study are openly available in the Genome Sequence Archive (GSA) at CRA019416.

## Supplementary Materials

The following supporting information can be downloaded at https://www.mdpi.com/article/10.3390/genes15111444/s1, Figure S1: Blood routine parameters not shown in the article; Table S1: qPCR primer sequences for DEGs in this article.

## References

[1] Aldemir H., Prod’homme V., Dumaurier M.-J., Retiere C., Poupon G., Cazareth J., Bihl F., Braud V.M. (2005). Cutting Edge: Lectin-Like Transcript 1 Is a Ligand for the CD161 Receptor. J. Immunol..

[2] Rosen D.B., Bettadapura J., Alsharifi M., Mathew P.A., Warren H.S., Lanier L.L. (2005). Cutting Edge: Lectin-Like Transcript-1 Is a Ligand for the Inhibitory Human NKR-P1A Receptor. J. Immunol..

[3] Boles K.S., Barten R., Kumaresan P.R., Trowsdale J., Mathew P.A. (1999). Cloning of a New Lectin-like Receptor Expressed on Human NK Cells. Immunogenetics.

[4] Braud V.M., Meghraoui-Kheddar A., Elaldi R., Petti L., Germain C., Anjuère F. (2022). LLT1-CD161 Interaction in Cancer: Promises and Challenges. Front. Immunol..

[5] Crunkhorn S. (2021). T Cell Atlas Reveals Route to Glioma Immunotherapy. Nat. Rev. Drug Discov..

[6] Tian Z., Chen Y., Gao B. (2013). Natural Killer Cells in Liver Disease. Hepatology.

[7] Liu P., Chen L., Zhang H. (2018). Natural Killer Cells in Liver Disease and Hepatocellular Carcinoma and the NK Cell-Based Immunotherapy. J. Immunol. Res..

[8] Kirkham C.L., Carlyle J.R. (2014). Complexity and Diversity of the NKR-P1: Clr (Klrb1: Clec2) Recognition Systems. Front. Immunol..

[9] Jameson G., Robinson M.W. (2021). Insights into Human Intrahepatic NK Cell Function From Single Cell RNA Sequencing Datasets. Front. Immunol..

[10] Li L., Hu Y., Li X., Ju B. (2024). A Comprehensive Analysis of the KLRB1 Expression and Its Clinical Implication in Testicular Germ Cell Tumors: A Review. Medicine.

[11] Chen Q., Yin H., Jiang Z., He T., Xie Y., Mao W., Han J., Liu S., Lou W., Wu W. (2024). Poor Clinical Outcomes and Immunoevasive Contexture in CD161+CD8+ T Cells Barren Human Pancreatic Cancer. J. Immunother. Cancer.

[12] Cha J., Kim D.H., Kim G., Cho J.W., Sung E., Baek S., Hong M.H., Kim C.G., Sim N.S., Hong H.J. (2024). Single-Cell Analysis Reveals Cellular and Molecular Factors Counteracting HPV-Positive Oropharyngeal Cancer Immunotherapy Outcomes. J. Immunother. Cancer.

[13] Qu J., Li Y., Wu B., Shen Q., Chen L., Sun W., Wang B., Ying L., Wu L., Zhou H. (2024). CD161+CD127+CD8+ T Cell Subsets Can Predict the Efficacy of Anti-PD-1 Immunotherapy in Non-Small Cell Lung Cancer with Diabetes Mellitus. OncoImmunology.

[14] Nasr Azadani H., Nassiri Toosi M., Shahmahmoodi S., Nejati A., Rahimi H., Farahmand M., Keshavarz A., Ghorbani Motlagh F., Samimi-Rad K. (2024). New Insights into Potential Biomarkers and Their Roles in Biological Processes Associated with Hepatitis C-Related Liver Cirrhosis by Hepatic RNA-Seq-Based Transcriptome Profiling. Virus Res..

[15] Fang S., Zhou Y. (2024). Deciphering the Role of KLRB1: A Novel Prognostic Indicator in Hepatocellular Carcinoma. BMC Gastroenterol..

[16] Mathewson N.D., Ashenberg O., Tirosh I., Gritsch S., Perez E.M., Marx S., Jerby-Arnon L., Chanoch-Myers R., Hara T., Richman A.R. (2021). Inhibitory CD161 Receptor Identified in Glioma-Infiltrating T Cells by Single-Cell Analysis. Cell.

[17] Sun J., Wang J., Zhang N., Yang R., Chen K., Kong D. (2019). Whole Transcriptome Analysis of Chemically Induced Hepatocellular Carcinoma Using RNA-Sequencing Analysis. FEBS Open Bio.

[18] Zhu M., Hao S., Liu T., Yang L., Zheng P., Zhang L., Ji G. (2017). Lingguizhugan Decoction Improves Non-Alcoholic Fatty Liver Disease by Altering Insulin Resistance and Lipid Metabolism Related Genes: A Whole Trancriptome Study by RNA-Seq. Oncotarget.

[19] Van Der Maaten L., Hinton G. (2008). Visualizing Data Using T-SNE. J. Mach. Learn. Res..

[20] Boettcher S., Manz M.G. (2017). Regulation of Inflammation- and Infection-Driven Hematopoiesis. Trends Immunol..

[21] Johnson C.B., Zhang J., Lucas D. (2020). The Role of the Bone Marrow Microenvironment in the Response to Infection. Front. Immunol..

[22] Freedman J.E., Loscalzo J. (2002). Platelet-Monocyte Aggregates: Bridging Thrombosis and Inflammation. Circulation.

[23] Hansen I.S., Baeten D.L.P., den Dunnen J. (2019). The Inflammatory Function of Human IgA. Cell. Mol. Life Sci..

[24] Elinav E., Strowig T., Henao-mejia J., Flavell R.A. (2011). Review Regulation of the Antimicrobial Response by NLR Proteins. Immunity.

[25] Linz B.M.L., Neely C.J., Kartchner L.B., Mendoza A.E., Khoury A.L., Truax A., Sempowski G., Eitas T., Brickey J., Ting J.P.Y. (2017). Innate Immune Cell Recovery Is Positively Regulated by NLRP12 during Emergency Hematopoiesis. J. Immunol..

[26] Garn H., Bahn S., Baune B.T., Binder E.B., Bisgaard H., Chatila T.A., Chavakis T., Culmsee C., Dannlowski U., Gay S. (2016). Current Concepts in Chronic Inflammatory Diseases: Interactions between Microbes, Cellular Metabolism, and Inflammation. J. Allergy Clin. Immunol..

[27] Fuentes E., Fuentes F., Vilahur G., Badimon L., Palomo I. (2013). Mechanisms of Chronic State of Inflammation as Mediators That Link Obese Adipose Tissue and Metabolic Syndrome. Mediat. Inflamm..

[28] Geissmann F., Manz M.G., Jung S., Sieweke M.H., Merad M., Ley K. (2010). Development of Monocytes, Macrophages, and Dendritic Cells. Science.

[29] Goto K., Morimoto M., Osaki M., Tanio A., Izutsu R., Fujiwara Y., Okada F. (2022). The Impact of AMIGO2 on Prognosis and Hepatic Metastasis in Gastric Cancer Patients. BMC Cancer.

[30] Bauernfeind A.L., Babbitt C.C. (2017). The Predictive Nature of Transcript Expression Levels on Protein Expression in Adult Human Brain. BMC Genom..

[31] Jiang A., Zhou Y., Gong W., Pan X., Gan X., Wu Z., Liu B., Qu L., Wang L. (2022). CCNA2 as an Immunological Biomarker Encompassing Tumor Microenvironment and Therapeutic Response in Multiple Cancer Types. Oxidative Med. Cell. Longev..

[32] Stangel D., Erkan M., Buchholz M., Gress T., Michalski C., Raulefs S., Friess H., Kleeff J. (2015). Kif20a Inhibition Reduces Migration and Invasion of Pancreatic Cancer Cells. J. Surg. Res..

[33] Gao W., Lu J., Yang Z., Li E., Cao Y., Xie L. (2024). Mitotic Functions and Characters of KIF11 in Cancers. Biomolecules.

[34] Li J., Gao J.Z., Du J.L., Huang Z.X., Wei L.X. (2014). Increased CDC20 Expression Is Associated with Development and Progression of Hepatocellular Carcinoma. Int. J. Oncol..

[35] Lv S., Xu W., Zhang Y., Zhang J., Dong X. (2020). NUF2 as an Anticancer Therapeutic Target and Prognostic Factor in Breast Cancer. Int. J. Oncol..

