# Supplementary figures and images for: Klrb1 Loss Promotes Chronic Hepatic Inflammation and Metabolic Dysregulation
Source: Genes (Basel). 2024 Nov 8;15(11):1444. doi: 10.3390/genes15111444 (PMC11594155; doi:10.3390/genes15111444)

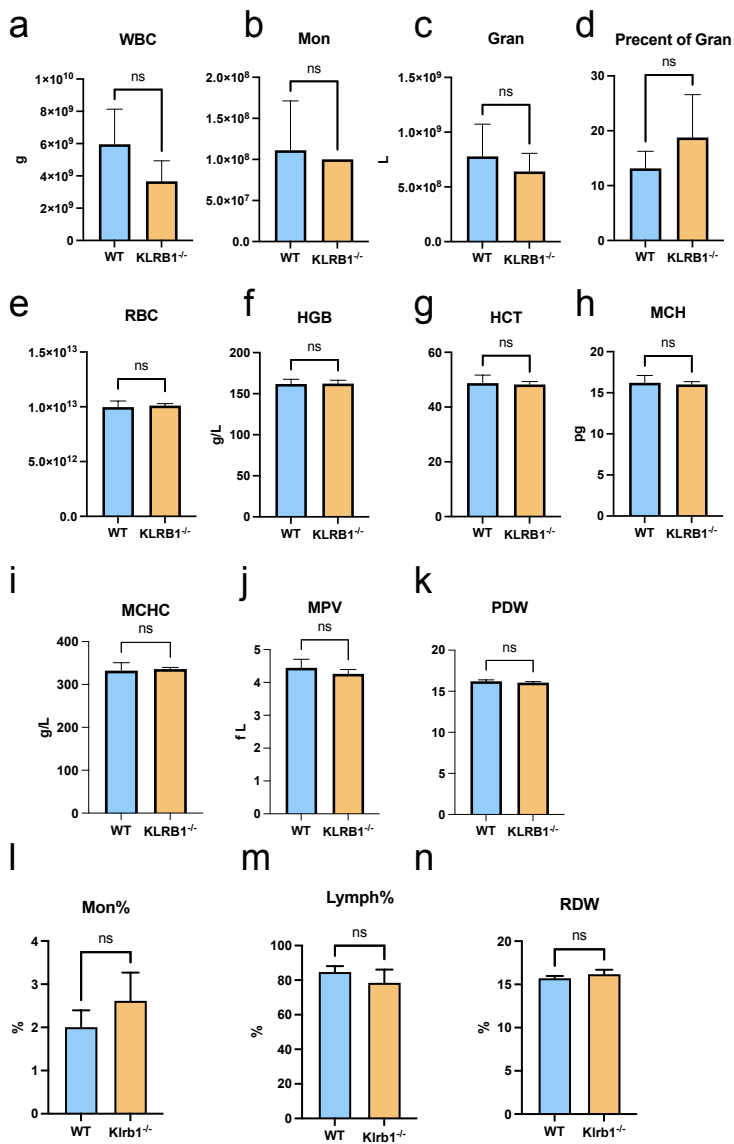

Supplement: Supplementary file 1 [file genes-15-01444-s001.zip › Figure S1.pdf]
